# The Necessity of Extensive Decompression for Spinal Epidural Hematoma: A Case Report and Literature Review

**DOI:** 10.7759/cureus.44192

**Published:** 2023-08-27

**Authors:** Mehmet Ali Kahraman, Salim Senturk

**Affiliations:** 1 Neurosurgery, Istanbul Medeniyet University, Prof. Dr. Suleyman Yalcin City Hospital, Istanbul, TUR; 2 Neurosurgery, Memorial Spinal Center, Istanbul, TUR

**Keywords:** lumbar pain, decompression, epidural hematoma, laminectomy, spinal hematoma

## Abstract

Spinal epidural hematomas (SEHs)are space-occupying lesions that exert pressure on the spinal cord by rapidly accumulating blood between the dura and bone or ligament components. The annual incidence of spontaneous epidural hematoma is estimated to be one in one million. The predominant symptoms are back pain or neurological impairment, including sensory, motor, or autonomic dysfunction of the limbs below the hematoma level. Depending on the level and size of the hematoma and the affected cord, they cause neurological deficits. Neurological deficits are often reversible if diagnosed and treated early with surgical decompression. However, neurological deficits can become permanent if the patient is not operated on timely, and paraplegia or quadriplegia may occur.

A 53-year-old man presented to our emergency department with acute-onset back pain and 36-hour-long, rapidly progressive paraparesis of both legs. On T1- and T2-weighted MRI scans, a hyperacute SEH was found as iso/hyperintense and hyperintense, respectively. Immediate decompressive laminectomy from T10 to L2 and hematoma evacuation were performed. It was challenging to remove the hematoma due to its firm consistency. Before performing a bilateral total laminectomy at five levels, the posterior spine was stabilized between T10 and L3 using transpedicular screws. Within 24 hours, the motor function of the lower limbs increased considerably. The patient could sit on a chair because of posterior stability.

In addition to the importance of early diagnosis using imaging techniques, planning the extension of SEH surgery is crucial for the patient’s postoperative neurological recovery.

## Introduction

Spinal epidural hematomas (SEHs) are space-occupying lesions that exert pressure on the spinal cord due to the fast accumulation of blood between the dura and bone or ligament components [1]. The etiology is attributed to various causes, and it can be iatrogenic following surgery or lumbar puncture, as well as traumatic [2] or spontaneous. Anti-coagulation or anti-aggregation therapy, as well as underlying coagulopathies, arteriovenous malformations, and hypertension, may be identified as the cause of SEHs, even though the disease’s primary predisposing factor cannot be determined in most cases.

The annual incidence of spontaneous epidural hematoma is one in one million [3]. Although it is a rare condition in the general population, it is far more common in neurosurgical practice, most likely due to iatrogenic causes. The predominant symptoms are back pain or neurological impairment, including sensory, motor, or autonomic dysfunction of the limbs below the hematoma level [4]. Spinal hematomas are most commonly located in the epidural space and have been reported to be six times more common than spinal subdural hematomas. SEHs are usually posterior to the spinal cord, while most spinal subdural hematomas are anterior to the spinal cord. Depending on the level of the affected cord, they present with pain and neurological deficits [5]. Small hematomas are managed conservatively. If large hematomas that have caused neurological deficits are diagnosed and treated early with surgical decompression, neurological deficits are often reversible. However, neurological deficits can become permanent if the patient is not operated on timely, and paraplegia or quadriplegia may occur [5,6].

MRI is the most reliable method for confirming the diagnosis and determining the hematoma’s extent. The patient may have a delayed or incorrect diagnosis as MRI is not the initial imaging modality. In addition to the significance of early diagnosis with imaging techniques, planning the extension of the operation is crucial for improving the patient’s neurological recovery after surgery [7].

Here, we present the case of a patient who underwent surgery for a massive SEH with substantial decompression and whose neurological evaluation exhibited simple immediate recovery.

## Case presentation

A 53-year-old man was admitted to the emergency department of our institution with acute-onset back pain for 36 hours and rapidly progressing paraparesis in both legs. There was no history of high-velocity trauma or aberrant motions. His medical history revealed hypertension and salicylic acid and clopidogrel use. The neurological test showed that the bilateral leg muscle strength was 1/5, and the patient was ASIA-B (American Spinal Injury Association Scale). Due to the weakening of his bladder functioning, he developed urine retention and required catheterization. The functions of the cranial nerves and cerebellum were normal. His vital signs were normal.

**Figure 1:**
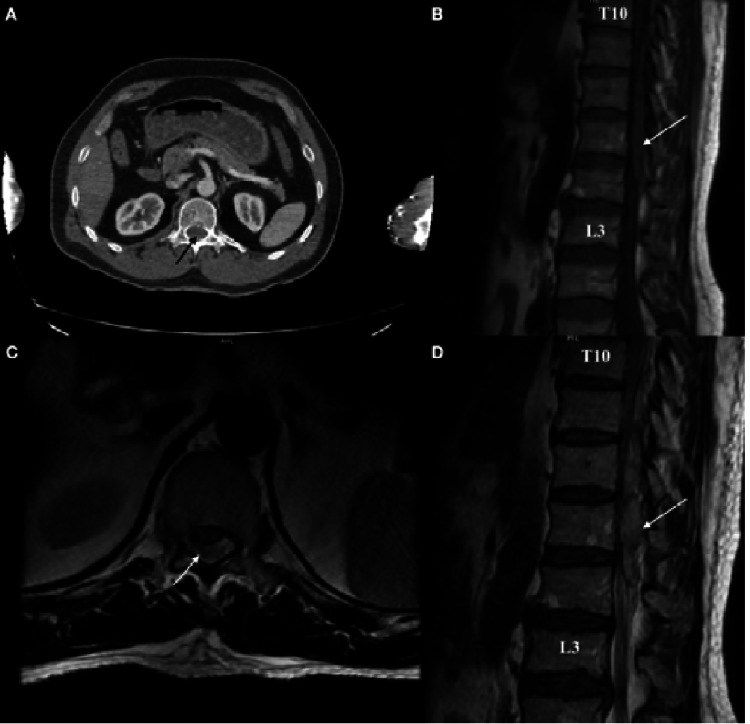
Preoperative images of the patient. A. CT image of isodense epidural hematoma located posterior to the spinal canal. B. T1-weighted sagittal MRI of iso/hypointense spinal epidural hemorrhage between the T10 and L2 levels. C. T2-weighted axial MRI of hyperintense spinal epidural hemorrhage located posterior to the spinal canal. D. T2-weighted axial MRI of hyperintense spinal epidural hemorrhage between the T10 and L2 levels. Arrows: spinal epidural hemorrhage.

A CT scan indicated no significant morphological alterations in the thoracic or lumbar vertebrae, apart from a suspiciously dense image surrounding the spinal cord. The thoracolumbar spine MRI showed posterior fluid collection, which appeared slightly isointense to the spinal cord on T1-weighted images, causing an external posterior mass effect on the dorsal surface dura mater and spinal cord displacement between T10 and L2 levels. The T2-weighted MRI images also showed hyperintensity (Figure 1). The definitive radiologic diagnosis was a hyperacute SEH.

**Figure 2:**
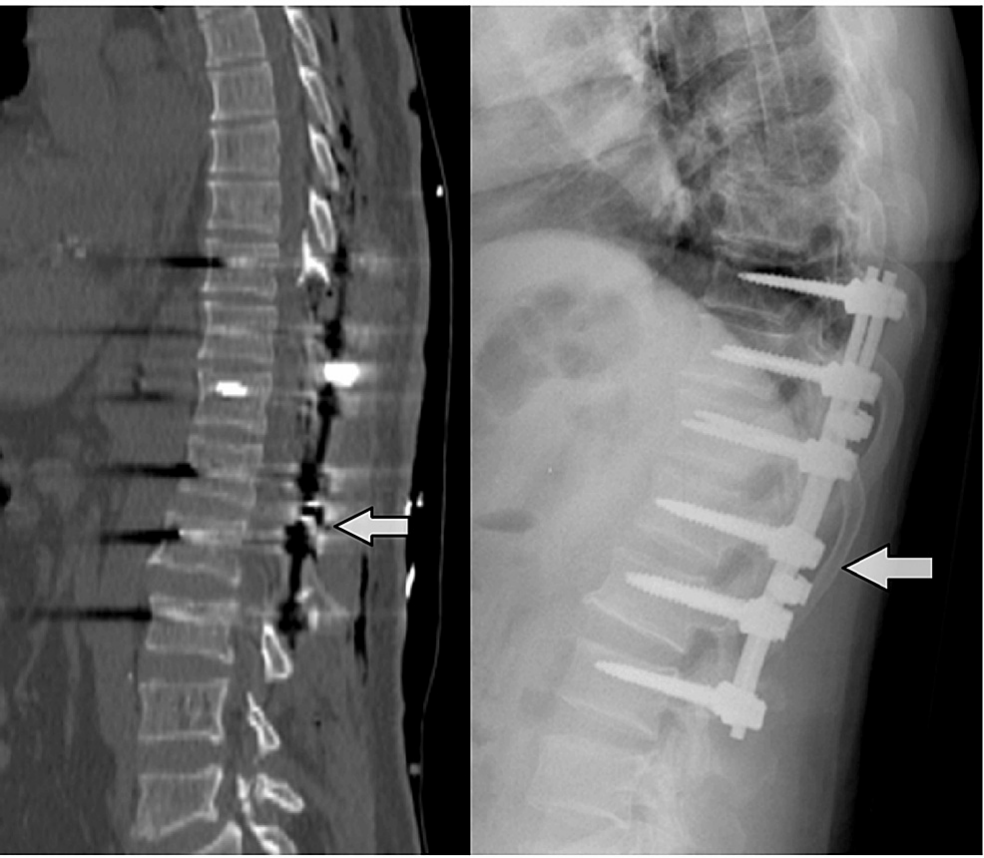
Postoperative images of the patient. A. Sagittal CT image because of five-level T10-L2 laminectomy. B. Lateral X-ray image showing T10-L3 posterior transpedicular stabilization. Arrows: operation field.

The operation was performed by a senior neurosurgeon as an emergency. The patient was immediately taken to the operation room for an emergency decompressive laminectomy from T10 to L2 and hematoma evacuation. Initially, a partial hemilaminectomy was performed. However, the hematoma could not be evacuated due to its hard structure. It was decided to conduct a comprehensive laminectomy to evacuate the hematoma and completely decompress the spinal cord (Figure 1). Before performing a total laminectomy at five levels, the posterior spine was stabilized between T10 and L3 using transpedicular screws. T10-L2 total laminectomy was performed following stabilization (Figure 2). The clot was positioned posteriorly, was well-organized, and could not be evacuated by aspiration alone. The disc punch and dissectors were utilized to remove the hematoma with a firm consistency.

The tissue was sent for histology after the hematoma was drained and the spinal cord was fully decompressed. No additional issues, such as vascular malformation, were observed during the operation. There was no apparent cause of bleeding, and hemostasis was achieved. The hematoma was confirmed by histology, and there was no evidence of abnormal blood vessels implying vascular or other abnormalities. Within 24 hours, the motor function of the lower limbs improved considerably. The bilateral leg motor force was at least 3/5. The patient could sit on a chair because of posterior stability. The patient was discharged without further complications three days following surgery. His bladder functions also returned to normal after a week of the surgery.

## Discussion

Jackson et al. initially characterized SEH as spinal apoplexy [5]. SEH might be spontaneous or caused by trauma. Changes in the coagulation cascade, vascular abnormalities, and pregnancy are the causes of spontaneous epidural bleeding. These can be observed following a spinal injury, iatrogenic injection, or surgery [6]. SEHs are rare in the human population, and following clinical suspicion, a proper and adequate clinical examination can lead to a diagnosis. It only accounts for 0.3% to 0.9% of all spinal canal lesions [7].

The causes of SEH can be classified as idiopathic, spontaneous, and secondary. The leading causes of secondary SEH are coagulopathies and anticoagulants [8,9], vascular abnormalities [10], tumors, and trauma [11]. The idiopathic reasons are epidural catheterization and lumbar surgery [12], with other risk factors including minor injuries and chiropractic manipulation [13,14]. Additional risk factors include pathologies of the bones and joints, such as Paget’s disease [15], ankylosing spondylitis [16], and rheumatoid arthritis [17]. Up to one-third of SEHs, however, lack an evident etiologic component. Even if a history of minor trauma is present, these are spontaneous SEHs.

Clinical signs of SEHs may be rapid and acute or develop gradually [18]. Differential diagnosis is required because disorders progressing over time can be confused with other space-occupying lesions, such as spinal tumors or infections [19]. In our case, the patient presented with acute-onset low back pain and bilateral limb paralysis that progressed rapidly. When he arrived at the hospital around 36 hours after the onset of symptoms, neither of his legs had any muscle strength. The rate of progression of clinical symptoms can be attributed to the canal’s diameter and the patient’s tolerance to the space-occupying effects of lumbar neurologic structures. Moreover, a more severe and quick neurological deterioration has been reported in cervical spine SEHs [20]. In our case, the hematoma was diagnosed between levels T10 and L2. Despite the wide diameter of the canal in the thoracolumbar and lumbar vertebrae, the spinal cord continues at these levels. This may explain why the neurological deterioration did not begin suddenly but advanced rapidly. The degeneration can be delayed in hematomas developed in the lower lumbar region.

Approximately 65% of SEHs are located behind multiple vertebrae. In 50% of instances, the hematoma is discovered behind three or more vertebrae [7]. The hematoma involving five vertebral segments was discovered in the spinal canal between T10 and L2 levels.

As the incidence and presentation of SEH symptoms vary from patient to patient, a differential diagnosis should be performed as soon as possible. Surgical intervention can result in morbidity, particularly in individuals whose clinical condition deteriorates fast due to a delayed diagnosis. In addition to back pain, a neurologic deficiency necessitates a radiographic assessment. Prompt imaging evaluation is required to determine the clinical degree of the lesion in accordance with the dermatomes and myotomes. Conventional radiographs are inadequate and unnecessary for neurologically deteriorating patients. For evaluating patients with spinal pathologies, CT is the first, quickest, most accessible, and least expensive radiological imaging technique. Although CT scans cannot diagnose hematoma directly, they can distinguish hyperacute hematoma from surrounding fat and bone structures [21]. MRI is the gold standard for imaging [22], but CT can be beneficial when MRI cannot be used or is contraindicated [23].

A subacute hematoma gradually becomes isodense on CT imaging, making it difficult to detect. MRI not only reveals the form of the hematoma but also provides a near-precise assessment of the cord levels it affects [24]. Moreover, it can provide information about the exact duration of bleeding. In the acute stage, hematoma appears hypointense on T1-weighted images and hyperintense on T2-weighted MRIs. Nevertheless, hyperacute hemorrhage appears iso-hypointense on T1-weighted images and hyperintense on T2-weighted MRIs. Early subacute hematoma is hyperintense on T1-weighted MRI and hypointense on T2-weighted images [22,25]. As time passes, the hematoma becomes more hyperintense on T1-weighted sequences and more hypointense on T2-weighted sequences. A mosaic pattern on T2-weighted MRI, which appears as a hyperintense signal with hypointense foci, has also been described as a distinctive characteristic [26]. To rule out causes such as vascular abnormalities, CT/MRI angiography may be performed preoperatively or postoperatively, depending on the amount of hematoma and its accessibility, to rule out potential causes. When CT or magnetic resonance angiography is questionable, digital subtraction angiography can also be performed. However, it is not required in emergencies [25].

In our case, the bleeding became isodense on CT imaging. This image suggests a space-occupying lesion, although it is not diagnostic of an epidural hematoma. MRI scans discovered a hematoma posterior to the spinal cord, which significantly compressed the spinal cord. On T1-weighted sequences, the hematoma was iso/hypointense; on T2-weighted sequences, it was hyperintense. These images indicate that the patient is experiencing hyperacute bleeding. The post-contrast series exhibited no contrast enhancement.

Although spontaneous recovery has been observed in patients with slow-onset symptoms and no neurological regression on evaluation [27,28], immediate surgical decompression and hematoma evacuation are suggested for symptomatic and rapidly progressive patients [29]. Surgical decompression typically involves laminectomy, hematoma evacuation, and hemostasis of any bleeding [30]. In most cases, early surgery can lead to better outcomes. In addition, the requirement of surgery for neurological recovery was emphasized in patients with severe or total deficits rather than leaving it to spontaneous resolution [29]. In cases with fast-progressing neurological symptoms, such as ours, laminectomy and hematoma evacuation successfully restore the neurological status. According to a case series, most SEH patients who received surgery 48 hours after the onset of symptoms had almost-complete neurologic recovery [31]. Another meta-analysis revealed that most individuals who received surgical therapy within 12 hours improved [32]. Like with other causes of spinal cord compression, the duration of compression is important in determining the likelihood of favorable results. Several published papers support decompression within varied time intervals, ranging from 12 hours to two days, as the best acceptable delay for satisfactory outcomes [29,33,34].

Although some surgeons have adopted minimally invasive laminectomy and tubular retractors in specific circumstances, their efficacy may be restricted to treating localized hematoma. It should be evaluated on a case-by-case basis [35]. While all studies have addressed the necessity and timing of surgery, the extent of surgery, whether minimally invasive or comprehensive laminectomy, still needs to be solved. Although the timing is crucial, the hematoma may already be coagulated and formed by the time the clinical course of a spinal cord injury has occurred. To effectively decompress a hematoma, direct access is required. Nevertheless, cord edema is a severe problem in secondary damage mechanisms, particularly in hematomas above L1-2, where the spinal cord continues. The pressure on the spinal cord causes the blood arteries that supply and drain the spinal cord to become compressed. In addition to interfering with vascular blood supply, it causes secondary harm due to biochemical and cellular processes [36]. When central necrosis and edema around the axons and myelin sheaths are caused by hemorrhage [37], laminectomy is recommended to identify and eliminate the hematoma, particularly after 36 hours when the hematoma is more intense and spinal cord edema can be alleviated. As previously stated, hematomas can occur at three levels and above. The hematoma, in our case, had extended to five vertebral levels. Surgical decompression of all levels is the only way to accomplish adequate decompression. Multilevel laminectomies are typically more unstable than single-level laminectomies; hence, posterior fixation and fusion are required following laminectomy [38]. Prevention of infection in long-level surgeries is also important for increasing morbidity [39].

Similarly, the patient’s postoperative clinical recovery was remarkable in our case as we could effectively decompress and remove the hematoma. Moreover, decompression minimized a further deficiency in the event of postoperative hemorrhage, as the area was previously prone to bleeding. Posterior stabilization was also performed during the same session, as the patient’s vital signs were stable, and he could undergo a lengthy operation. Hence, with the assistance of early mobilization, the patient could submit an early application for physical therapy and rehabilitation.

## Conclusions

Comprehensive planning of the extension of SEH surgery is crucial, in addition to the importance of early diagnosis using imaging techniques. The extension of SEH surgery is significant for the patient’s postoperative neurological recovery.
